# Treatment of Hydrocele

**Published:** 1847-04

**Authors:** 


*Treatment of Hydrocele.*—In the treatment of hydrocele, M. Blandin does not believe that the nature of the liquid injected into the vaginal tunic exercises any sensible influence upon the results of the operation. He employs, with equal confidence, the vinous decoction of roses de Provins, tincture of iodine, etc. It is only necessary that the liquid should possess irritating properties, in a sufficient degree to provoke an adhesive inflammation, in order that its use may be followed by success. He prefers a simple mixture of three parts of water to one of rectified alcohol. This liquid possesses the advantage of being always at the hand of the surgeon, and not requiring, as the vinous decoction does, a manipulation more or less tedious; and what is of more importance, it possesses over the preparations of iodine the advantage of leaving no stain on the instruments or on the linen of the patients.—*Ibid.*

